# Third lumbar transverse process syndrome with acute abdominal pain as the main clinical manifestation: A case report

**DOI:** 10.1097/MD.0000000000051067

**Published:** 2026-10-16

**Authors:** Guangcheng Wei, Ningjuan Yan, Guodong Zhao, Tao Liu, Ming Chen, Weikai Qin, Yong Zhao

**Affiliations:** a Sports Medicine Department, Wangjing Hospital, China Academy of China Medical Sciences, Chaoyang District, Beijing, China; b Department of Spleen and Stomach Diseases, Wangjing Hospital, China Academy of Chinese Medical Sciences, Chaoyang District, Beijing, China.

**Keywords:** acupotomy, case report, lumbar vertebrae, manipulation, spinal-origin abdominal pain, third lumbar transverse process syndrome

## Abstract

**Rationale:**

Third lumbar transverse process syndrome is an uncommon musculoskeletal cause of referred abdominal pain. Because acute abdominal pain is appropriately evaluated first for visceral, vascular, urinary, and surgical emergencies, a spinal or paraspinal pain generator may be overlooked.

**Patient concerns:**

A 66-year-old man presented with persistent left lower-quadrant abdominal pain radiating to the left lower back. Baseline pain intensity was 7/10 on a VAS, and the pain was aggravated by postural change, standing, and walking.

**Diagnoses:**

Serial laboratory tests, endoscopy, ultrasonography, CT/CTA, and specialist consultations did not identify a visceral, urinary, surgical, or hemodynamically significant vascular disorder sufficient to explain the pain. Targeted musculoskeletal examination showed focal tenderness and increased soft-tissue tension around the left L3 transverse process, movement-related pain, and a visually estimated compensatory apparent leg-length discrepancy. These findings supported a clinical diagnosis of third lumbar transverse process syndrome presenting as referred abdominal pain.

**Interventions:**

The patient received Tuina manual therapy, two acupotomy closed-release procedures at palpation-defined tender points in the left lumbar and gluteal regions, and adjunctive pharmacotherapy.

**Outcomes:**

At discharge on February 9, 2021, the abdomen was soft without tenderness or rebound tenderness. Abdominal pain had resolved completely at the 2-week follow-up. Routine annual telephone follow-up of former inpatients was then conducted once yearly for 3 years, with no recurrence reported.

**Lessons:**

After urgent abdominal and vascular disorders have been excluded, history taking and targeted examination of the lumbar transverse processes may identify a musculoskeletal source of otherwise unexplained abdominal pain.

## 1. Introduction

Third lumbar transverse process syndrome is an uncommon musculoskeletal disorder that can produce low-back pain and referred symptoms. Spinal-origin abdominal pain has been associated with vertebral fracture, neoplasia, spinal infection, stenosis, disc disease, paraspinal lesions, and lumbar myofascial disorders.^[1,2]^ Mechanical loading, local inflammation, chemical mediators, and neural convergence may contribute to symptom generation.

The L3 transverse process is relatively long and provides attachment for the quadratus lumborum, psoas major, intertransversarii, thoracolumbar fascia, and associated tendinous structures. L3 also lies near the center of lumbar motion. Repetitive traction and shear at these attachments may produce local enthesopathic or myofascial injury, inflammation, and referred pain. Functional continuity between the thoracolumbar fascia, paraspinal musculature, and abdominal wall offers a plausible anatomical basis for abdominal symptoms.^[3,4]^

Clinicians evaluating acute abdominal pain appropriately prioritize visceral, vascular, urinary, and surgical emergencies, and spinal or paraspinal dysfunction may not be included in the initial differential diagnosis. Moreover, routine lumbar imaging may show nonspecific degenerative changes while failing to characterize a clinically painful soft-tissue lesion.^[4–6]^

This case report describes the diagnostic sequence, documented treatment, inpatient course, and reported follow-up of third lumbar transverse process syndrome presenting predominantly as acute abdominal pain. It highlights the role of focused musculoskeletal examination after urgent abdominal and vascular causes have been excluded.

## 2. Case description

### 2.1. Patient information and initial presentation

A 66-year-old man developed left lower abdominal distension and pain without an obvious precipitating event on the evening of December 21, 2020. The pain radiated to the left lower back and was aggravated by postural change, standing, and walking. His medical history included mitral valve repair, radiofrequency catheter ablation for paroxysmal atrial fibrillation, and remote urolithiasis. He had no fever, hematemesis, hematochezia, or melena.

On December 22, 2020, he attended Hospital A. His heart rate was 84 beats/min, body temperature 36.3°C, blood pressure 175/70 mm Hg, and bowel sounds 4/min. Examination showed left lower-quadrant tenderness without rebound tenderness or guarding. A complete blood count, serum biochemistry including liver and kidney function and amylase, electrocardiography, abdominopelvic CT, and CTA did not show an acute abdominal lesion. CTA was reported as showing approximately 60% proximal IMA stenosis (Fig. 1). Because abdominal distension and constipation were prominent, an antispasmodic and an enema were administered symptomatically; relief was transient.

**Figure 1.**
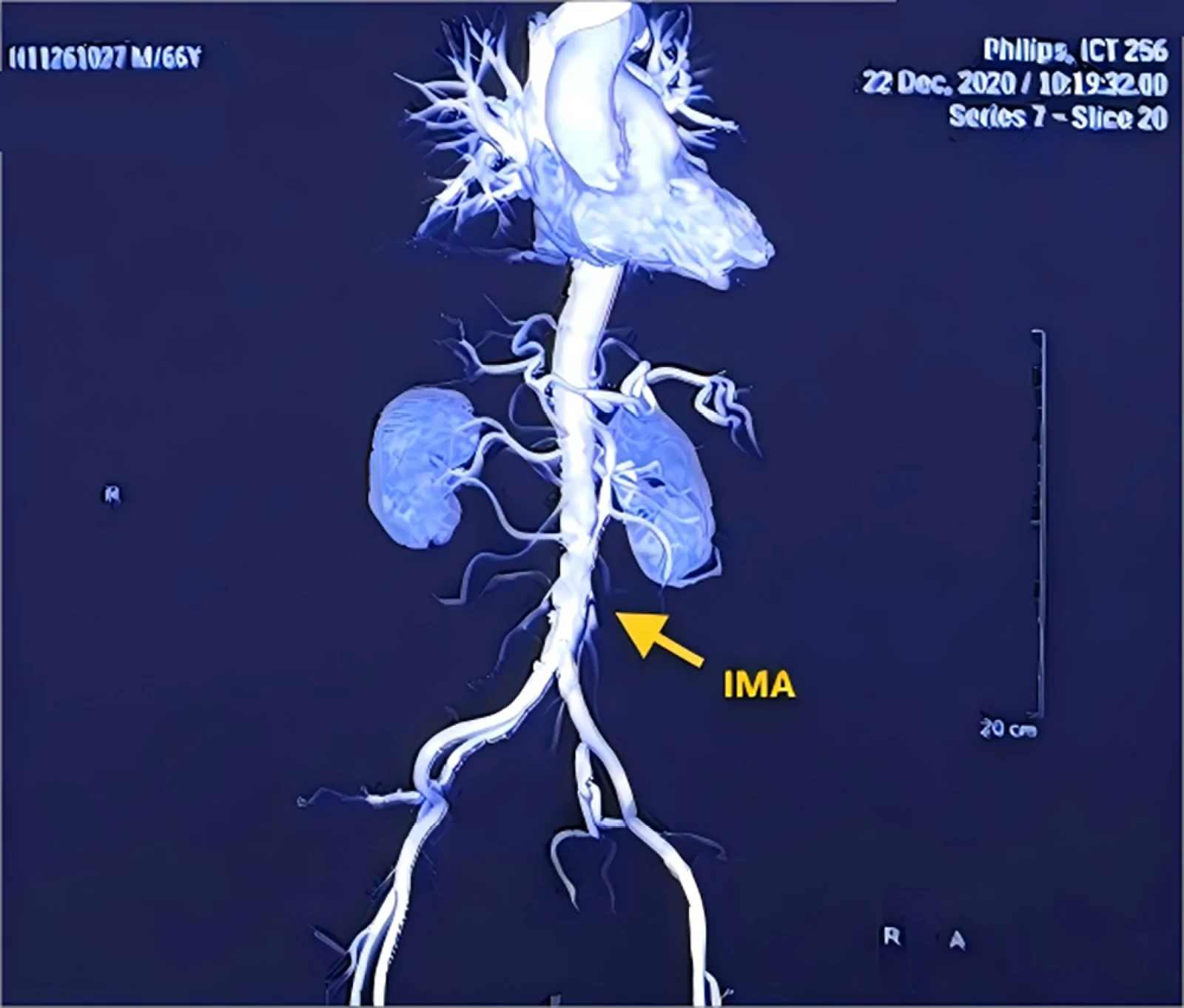
Aortic CTA: Moderate stenosis at the proximal segment of the IMA. 3D CT angiography demonstrates branching vessels in the abdominal aorta. The inferior mesenteric artery (IMA) was annotated in the image. Imaging parameters: Philips ICT 256 scanner with 1 mm slice thickness, contrast agent administered at a flow rate of 3.5 mL/s. Patient identifiers were anonymized.

Later on December 22, 2020, he attended the emergency department of Hospital B. Baseline pain intensity was documented as 7/10 on a VAS. The ensuing evaluation from December 22 to 31 comprised serial complete blood counts with differential; hsCRP and PCT; prothrombin time, international normalized ratio, activated partial thromboplastin time, fibrinogen, and D-dimer; serum hepatic and renal indices, electrolytes, glucose, creatine kinase, CK-MB, cardiac troponin I, and myoglobin; serial urinalysis; fecal microscopy and occult-blood testing; fecal fungal smear, acid-fast staining, and bacterial and fungal cultures; contrast-enhanced abdominopelvic CT/CTA; hepatobiliary, pancreatic, renal, ureteral, and bladder ultrasonography; prostate and scrotal ultrasonography; mesenteric Doppler ultrasonography; and lumbar MRI (Fig. 2). General surgical, vascular surgical, urological, and orthopedic consultations did not identify gastrointestinal perforation or necrosis, urinary calculus, clinically significant mesenteric ischemia, or another urgent visceral disorder sufficient to explain the pain. Symptomatic antispasmodic, analgesic, and antibiotic treatment did not provide sustained relief.

**Figure 2.**
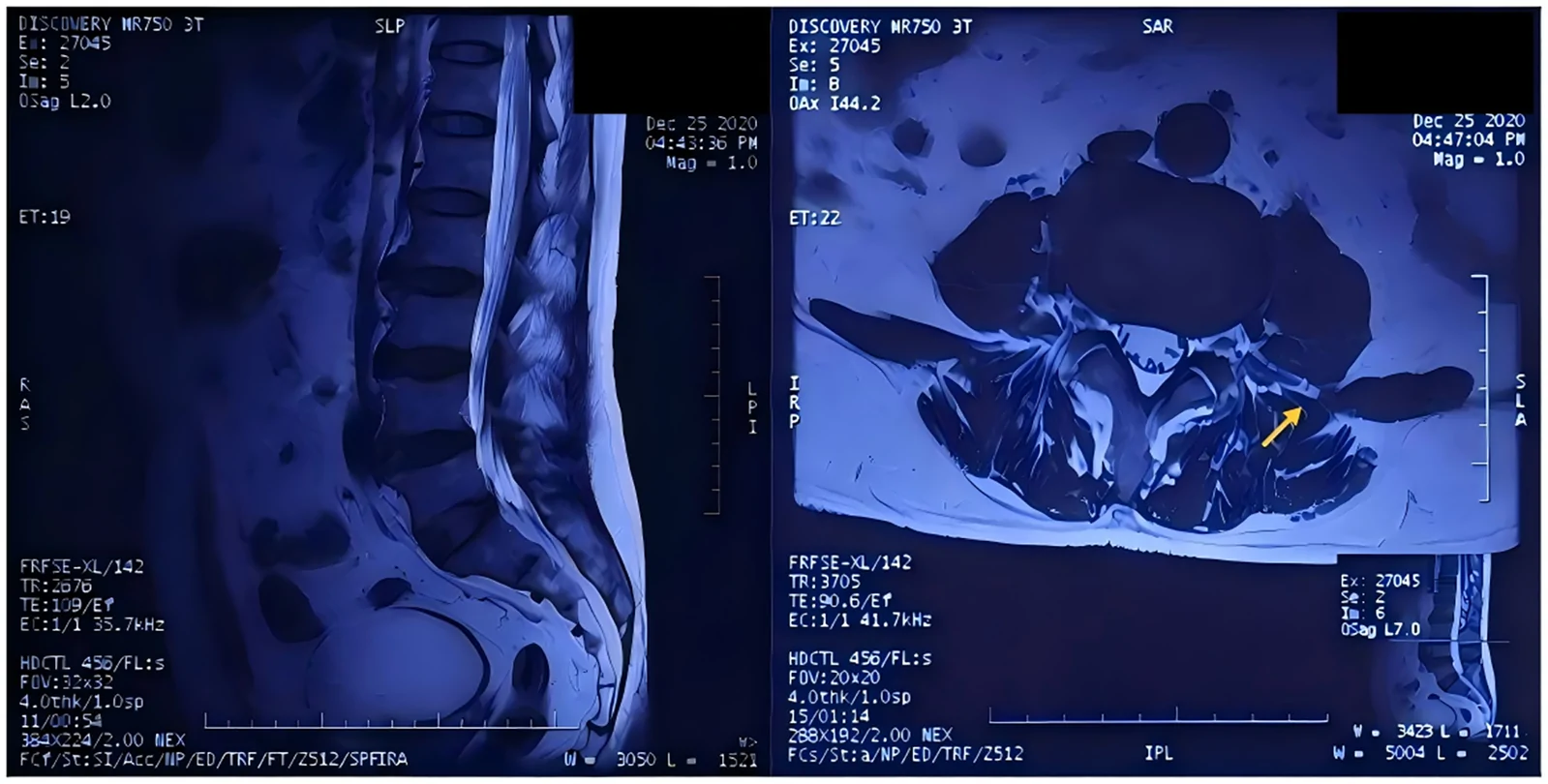
Lumbar MR (T2WI): degenerative changes, with disc degeneration and mild disc bulging from L2 to L5.

He was evaluated at Wangjing Hospital on December 30, 2020. Colonoscopy on January 4, 2021, showed multiple colonic polyps. He was admitted to the Department of Spleen and Stomach Diseases at 15:52 on January 5, 2021, for persistent left lower abdominal pain and further management of the polyps. On admission, body temperature was 36.6°C, pulse rate 76 beats/min, respiratory rate 18 breaths/m, and blood pressure 150/76 mm Hg. The abdomen was soft with focal left lower-quadrant tenderness and no rebound tenderness.

### 2.2. Clinical findings and targeted musculoskeletal assessment

On January 6, 2021, orthopedic consultation identified localized tenderness, increased soft-tissue tension around the left L3 transverse process, and positive paraspinal percussion and cough-provocation findings. Associate Chief Physician Guodong Zhao visually estimated that the left lower limb appeared approximately 1 cm longer than the right. This was interpreted as a compensatory apparent leg-length discrepancy.

### 2.3. Diagnostic assessment

At Hospital B, hsCRP was 88.41 mg/L on December 23 and 103.03 mg/L on December 24. PCT was 0.33 ng/mL and D-dimer 0.91 mg/L FEU on December 23. CT/CTA demonstrated abdominal aortic atherosclerosis, mild SMA stenosis, and focal proximal IMA stenosis with preserved distal opacification, without an acute abdominal lesion sufficient to explain the pain. Mesenteric ultrasonography on December 25 showed patent visualized SMA and IMA flow with PSVs of 122 and 133 cm/s, respectively; the SMV was patent, and the inferior mesenteric vein was poorly visualized. Vascular consultation concluded that the IMA stenosis was unlikely to explain the symptoms. Lumbar MRI on December 25 showed straightening of lumbar lordosis, L2-L5 disc degeneration, and mild disc bulging, without lateral recess stenosis or abnormal spinal cord signal. The initial orthopedic assessment did not identify these nonspecific degenerative findings as the cause of abdominal pain.

At Wangjing Hospital, chest CT on December 30 showed bronchitic and emphysematous changes, mild bilateral lower-lobe interstitial change, old pulmonary lesions, patchy infectious-appearing opacities in the posterior right lung, and right pleural thickening and calcification with loculated effusion. On the same date, hsCRP had decreased to 17.2 mg/L, the white blood cell count was 5.03 × 10^9^/L, neutrophils 77.9%, hemoglobin 135 g/L, and platelets 207 × 10^9^/L. Colonoscopy on January 4 showed multiple colonic polyps. Lumbar spine radiographs obtained on January 6 showed lumbar scoliosis and degenerative changes (Fig. 3). Key investigations are summarized in Table 1.

**Table 1:** Key laboratory and imaging findings.

Date	Institution	Investigation	Principal result
Dec 22, 2020	Hospital A	CBC with differential; hepatic and renal biochemistry; GGT; amylase; D-dimer; ECG; abdominopelvic CT	WBC 7.48 × 10^9^/L; neutrophils 76.3%; D-dimer 1.30 mg/L; GGT 138.50 U/L. Amylase, ECG, and CT showed no acute abnormality sufficient to explain the pain.
Dec 22, 2020	Hospital A	Contrast-enhanced CT/CTA	Approximately 60% proximal IMA stenosis was reported.
Dec 23, 2020	Hospital B	CBC with differential; hsCRP; PCT; coagulation including D-dimer; hepatic/renal biochemistry; electrolytes; glucose; cardiac biomarkers	WBC 7.70 × 10^9^/L; neutrophils 77.0%; hemoglobin 132 g/L; platelets 169 × 10^9^/L; hsCRP 88.41 mg/L; PCT 0.33 ng/mL; D-dimer 0.91 mg/L FEU; creatinine 77 μmol/L. No explanatory hepatic, renal, electrolyte, or cardiac-biomarker abnormality.
Dec 24, 2020	Hospital B	CBC with differential and hsCRP	WBC 6.91 × 10^9^/L; neutrophils 76.1%; hemoglobin 138 g/L; platelets 187 × 10^9^/L; hsCRP 103.03 mg/L.
Dec 22–24, 2020	Hospital B	Serial urinalysis	Leukocytes and nitrite negative. Transient trace to 0.3 g/L protein, trace blood, ketonuria up to 1.5 mmol/L, and glycosuria 5.5 mmol/L on Dec 22.
Dec 23, 2020 (reported Dec 28)	Hospital B	Contrast-enhanced abdominopelvic CT/CTA	Aortic atherosclerosis; mild SMA stenosis; focal proximal IMA stenosis with preserved distal filling; no acute abdominal cause. Incidental right loculated pleural effusion with pleural thickening/calcification, mild colonic gas distension, pancreatic fatty infiltration, prostate calcification, and thoracolumbar osteophytes.
Dec 24, 2020	Hospital B	Renal, ureteral, bladder, and prostate ultrasonography	No hydronephrosis, ureteral dilatation, or definite urinary calculus. Prostate approximately 5.0 × 5.6 × 4.5 cm, protruding 1.4 cm into the bladder.
Dec 23, 2020	Hospital B	Scrotal ultrasonography	A 0.4-cm left intratesticular anechoic lesion compatible with a cyst; no abnormal testicular blood-flow signal.
Dec 25, 2020	Hospital B	Mesenteric Doppler ultrasonography	SMA PSV 122 cm/s; visible IMA lumen approximately 0.22 cm with PSV 133 cm/s and patent flow; SMV patent; inferior mesenteric vein poorly visualized.
Dec 25, 2020	Hospital B	Lumbar MRI	Straightened lumbar lordosis; L2-L5 disc degeneration and mild bulging; slight L2-L3 and L4-L5 disc-space narrowing; no lateral recess stenosis or abnormal spinal cord signal.
Dec 29, 2020	Hospital B	Fecal microscopy and occult-blood testing	Yellow loose stool; 5–7 leukocytes/high-power field; no erythrocytes; occult blood positive.
Dec 31, 2020	Hospital B	Fecal fungal smear, acid-fast stain, and bacterial/fungal cultures	Fungal smear and acid-fast stain negative; Salmonella and Shigella showed no growth; Candida glabrata cultured (semiquantitative growth, ++).
Dec 30, 2020	Wangjing Hospital	Chest CT	Bronchitic and emphysematous changes; mild bilateral lower-lobe interstitial change; old pulmonary lesions; posterior right-lung infectious-appearing opacities; right pleural thickening/calcification with loculated effusion.
Dec 30, 2020	Wangjing Hospital	CBC with differential and hsCRP	WBC 5.03 × 10^9^/L; neutrophils 77.9%; lymphocytes 13.5%; hemoglobin 135 g/L; platelets 207 × 10^9^/L; hsCRP 17.2 mg/L.
Jan 4, 2021	Wangjing Hospital	Colonoscopy	Multiple colonic polyps; no colonoscopic finding explained the position-dependent pain.
Jan 6, 2021	Wangjing Hospital	Lumbar spine radiography	Lumbar scoliosis and degenerative changes.

CBC = complete blood count, CK-MB = creatine kinase-MB, CTA = computed tomography angiography, ECG = electrocardiography, GGT = gamma-glutamyl transferase, hsCRP = high-sensitivity C-reactive protein, IMA = inferior mesenteric artery, MRI = magnetic resonance imaging, PCT = procalcitonin, PSV = peak systolic velocity, SMA = superior mesenteric artery, SMV = superior mesenteric vein, WBC = white blood cell count.

**Figure 3.**
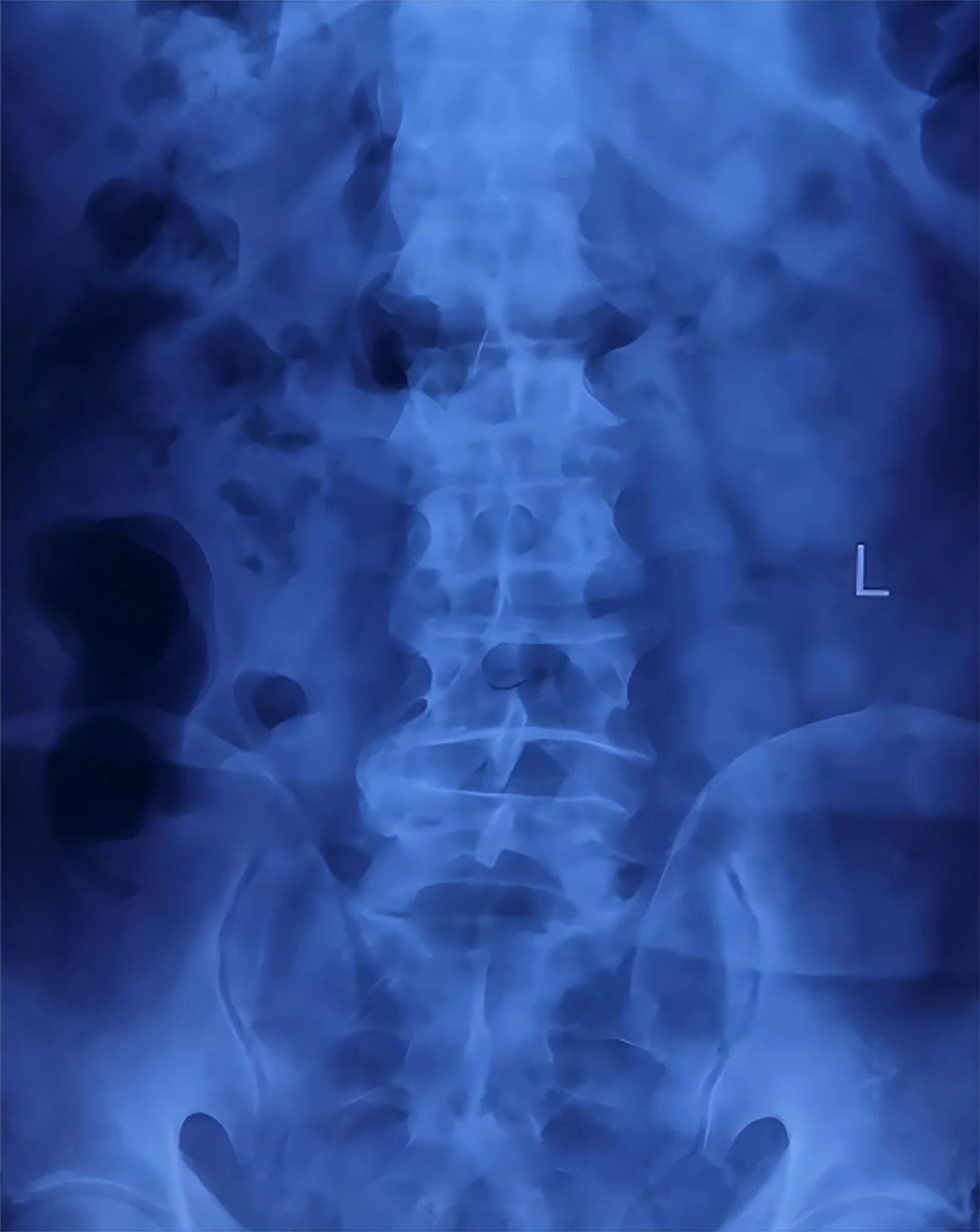
Lumbar Spine X-ray: lumbar scoliosis and degenerative changes.

No visceral, urinary, surgical, or hemodynamically significant vascular disorder was identified that adequately explained the severe position-dependent pain. The combination of pain radiating to the lower back, movement-related aggravation, focal L3 transverse-process tenderness and adjacent soft-tissue tension, positive paraspinal percussion and cough-provocation findings, and exclusion of urgent abdominal causes supported a clinical diagnosis of third lumbar transverse process syndrome presenting as referred abdominal pain.

### 2.4. Therapeutic interventions

The patient was transferred to the Sports Medicine Department on January 7, 2021. The documented treatment was multimodal.

Tuina was performed by Associate Chief Physician Guodong Zhao in three sequential stages. First, with the patient prone, the soft tissues surrounding the left L3 transverse process were treated using rolling manipulation (gun fa, Fig. 4A) and transverse plucking manipulation (bo fa, Fig. 4B). Rolling was applied over the left lumbar paraspinal muscles, followed by controlled plucking across the taut and tender soft tissues adjacent to the L3 transverse process. The intensity was gradually increased within the patient’s tolerance to reduce local muscular tension. Second, the three-step lumbar pulling manipulation (san ban fa) was performed. During the leg-lifting and lumbar-pushing maneuver, the ipsilateral thigh was elevated while the affected lumbar region was similarly pushed and released 3 times (Fig. 5A). During the shoulder-lifting and back-pushing maneuver, the patient remained prone while the shoulder was gently elevated and the symptomatic lumbar region was rhythmically pushed and released 3 times in a bow-like motion (Fig. 5B). During the shoulder-lifting and hip-pushing maneuver, the patient was placed in the lateral decubitus position with the upper hip and knee flexed and the lower limb extended. The shoulder was drawn posteriorly while the pelvis was pushed anteriorly (Fig. 5C). After several gentle mobilizations, the rotational range was gradually increased, and a brief, controlled impulse was delivered when a firm end point was reached. Third, the medial-hip alignment maneuver (kuan nei ce shoufa) was performed with the patient supine and the clinician standing on the affected side. One hand grasped the ankle while the other supported the lower limb. Under longitudinal traction, the affected lower limb was gently oscillated through a circular arc 6 to 7 times. The supporting hand was then repositioned over the knee; the hip and knee were flexed, and controlled downward pressure was applied. The hand at the ankle was adjusted so that the thumb contacted the posteroinferior aspect of the ischial tuberosity (Fig. 6A). The other hand subsequently grasped the distal lower leg and guided the affected limb into extension while coordinated counterpressure was applied with the thumb at the ischial tuberosity (Fig. 6B). The maneuver was concluded after apparent lower-limb symmetry had been visually reassessed. All manipulations were performed within the patient’s tolerance.^[7]^

**Figure 4.**
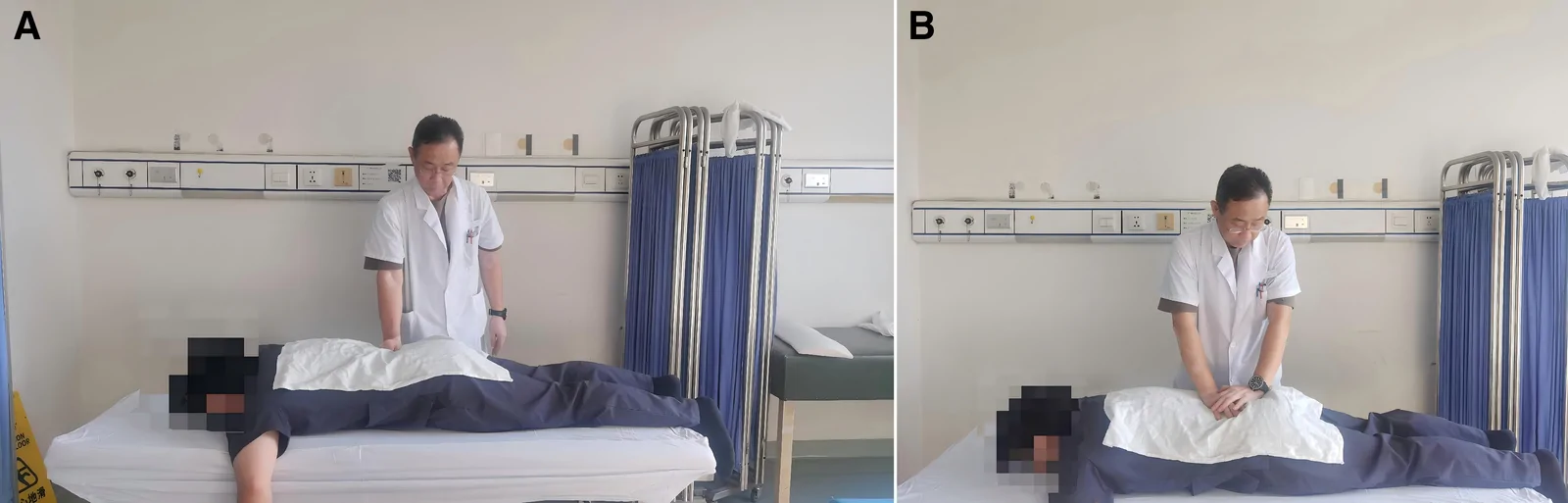
(A) Release Maneuvers: Rolling Manipulation (gun fa). (B) Release Maneuvers: Transverse Plucking Manipulation (bo fa).

**Figure 5.**
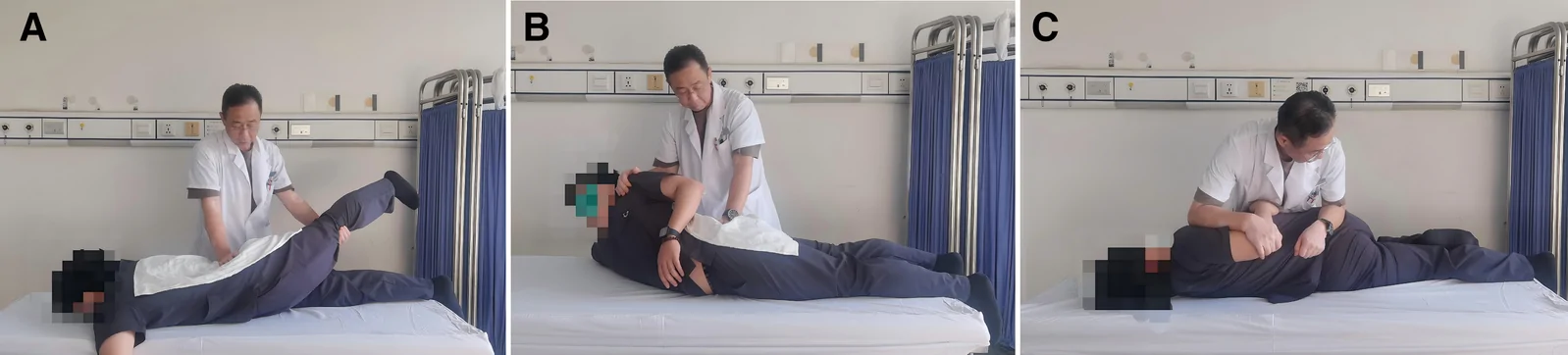
(A) The three-step Lumbar Pulling Manipulation (san ban fa): Leg Pulling Manipulation. (B) The three-step Lumbar Pulling Manipulation (san ban fa): Shoulder Pulling Manipulation. (C) The three-step Lumbar Pulling Manipulation (san ban fa): Oblique Pulling Manipulation.

**Figure 6.**
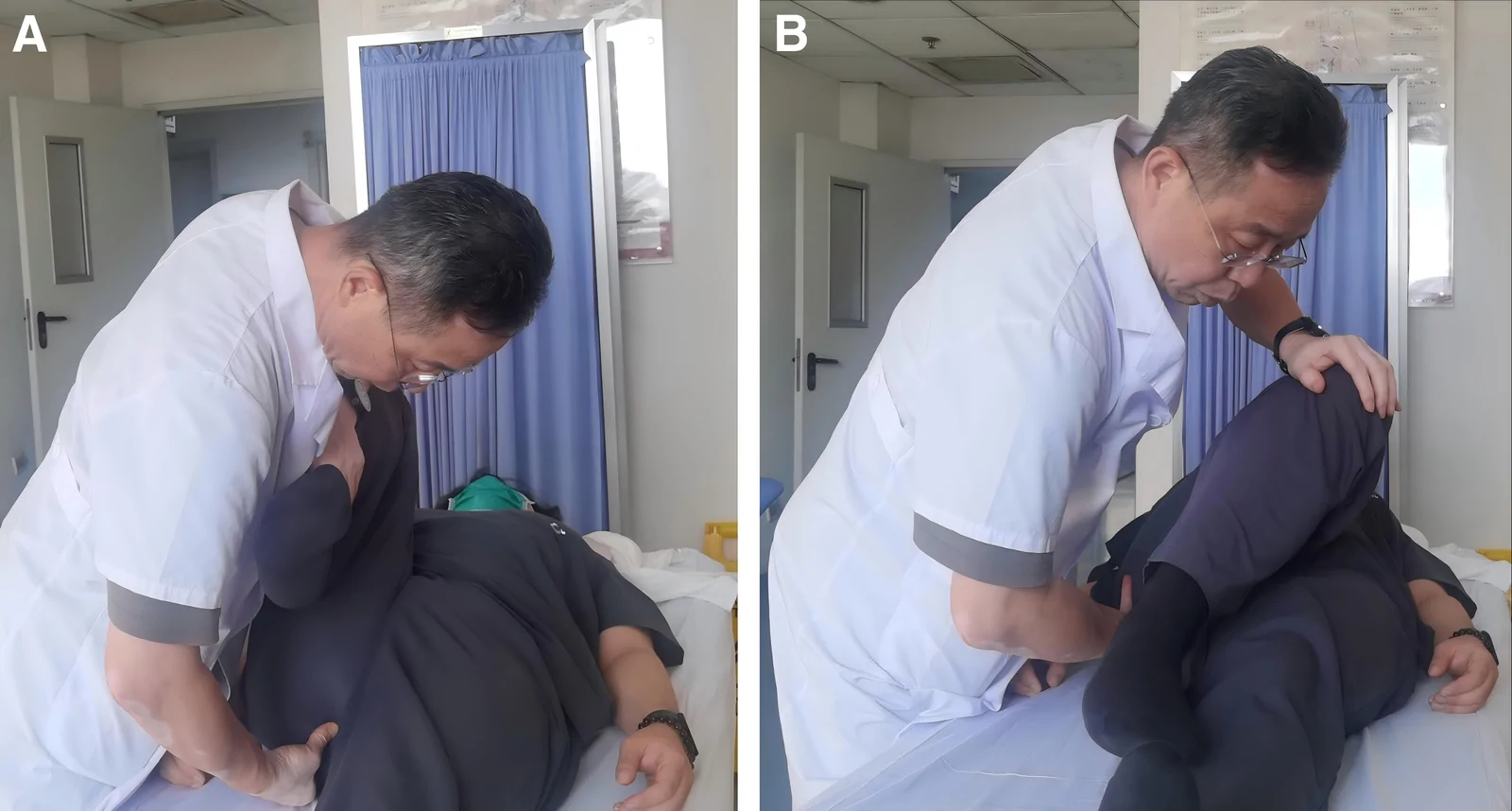
(A) The Medial-hip Alignment Maneuver (kuan nei ce shoufa). (B) The Medial-hip Alignment Maneuver (kuan nei ce shoufa).

Two acupotomy closed-release procedures are documented. A 0.8 × 40-mm disposable sterile acupotomy instrument manufactured by Jiangsu Huayou Medical Devices Co., Ltd. (Chinese medical device registration No. Su Xie Zhu Zhun 20172270978 [苏械注准20172270978]) was used. On January 13 and January 18, 2021, palpation-defined tender points around the left L3 transverse process and in the left gluteal region were identified and marked with the patient supine. After routine skin disinfection, the operator advanced the instrument rapidly through the skin to the fascial layer and released the thickened fascia with several passes. The notes describe palpable or audible tissue-release sounds. The instrument was removed and a sterile dressing was applied.

Adjunctive medication during the orthopedic admission included adenosylcobalamin 1.5 mg intramuscularly once daily, 0.9g intravenous lysine acetylsalicylate once daily, and Danhong injection 40 mL intravenously once daily.

### 2.5. Inpatient course, outcomes, and follow-up

On January 6, following the orthopedic consultation and Tuina treatment, the patient’s abdominal pain was markedly alleviated, and he was able to sit upright and ambulate slowly. However, the abdominal pain partially recurred that evening.

On January 13, before the first acupotomy procedure, L3 transverse-process tenderness and adjacent percussion pain had decreased, and the cough-provocation sign was negative. On January 16, lumbar pain was substantially reduced, and the patient could walk slowly.

After the second acupotomy procedure on January 18, lumbar pain and L3 tenderness were reduced, paraspinal percussion pain and the cough-provocation sign were negative, and no immediate procedure-related adverse event was recorded. The patient was transferred back to the Department of Spleen and Stomach Diseases for gastrointestinal management. On January 20, colonoscopy with EMR and APC was performed for multiple colonic polyps. He remained continuously hospitalized from January 5 through February 9, 2021, for a total of 35 days.

At discharge on February 9, the abdomen was soft without tenderness or rebound tenderness. Mild pain remained in the left inguinal and hip regions, but mobility was restored. Abdominal pain had resolved completely at the 2-week follow-up. Thereafter, routine annual telephone follow-up of former inpatients was conducted once yearly for 3 years, and no recurrence was reported.

Timeline of the patient’s clinical course is summarized in Table 2.

**Table 2:** Timeline of the patient’s clinical course.

Date	Setting	Key finding	Intervention/outcome
Dec 21, 2020	Symptom onset	Left lower-quadrant pain radiating to the left lower back; movement-related aggravation.	No precipitating event identified.
Dec 22, 2020	Hospital A	Left lower-quadrant tenderness without peritoneal signs; CT showed no acute explanatory lesion; CTA reported approximately 60% proximal IMA stenosis.	Antispasmodic and enema for distension/constipation; transient relief.
Dec 22–25, 2020	Hospital B	VAS 7/10; serial hematology, inflammatory-marker, coagulation, biochemical, urinalysis, and fecal studies; enhanced CT/CTA; hepatobiliary-pancreatic, urinary, prostate, scrotal, and mesenteric ultrasonography; lumbar MRI; and general surgical, vascular surgical, urological, and orthopedic consultations. No surgical, urinary, or hemodynamically significant vascular emergency was identified.	Symptomatic treatment; pain persisted.
Dec 30, 2020–Jan 5, 2021	Wangjing Hospital	hsCRP decreased; colonoscopy Jan 4 showed multiple colonic polyps; admitted Jan 5 for persistent pain and polyp management.	Gastrointestinal evaluation and symptomatic care.
Jan 6–7, 2021	Sports Medicine Department consultation/transfer	Lumbar radiography showed scoliosis and degenerative changes; focal left L3 tenderness and soft-tissue tension were present, with a visually estimated compensatory apparent leg-length discrepancy; third lumbar transverse process syndrome was considered.	Transferred to Orthopedics and Traumatology. Tuina after consultation markedly reduced abdominal pain and permitted sitting and slow ambulation; pain partially recurred that evening.
Jan 13, 2021	Sports Medicine Department ward	L3 findings improved; cough-provocation sign negative.	First documented needle-knife closed release.
Jan 18, 2021	Sports Medicine Department ward/transfer	Lumbar pain and focal findings improved; no immediate acupotomy adverse event.	Second documented needle-knife closed release; transferred back for gastrointestinal care.
Jan 20, 2021	Spleen and Stomach Diseases	Multiple colonic polyps treated endoscopically.	Colonoscopy with EMR and APC; postprocedural care.
Feb 9, 2021	Discharge	Abdomen soft and nontender; mild left inguinal/hip pain; mobility restored.	Discharged after continuous 35-day hospitalization.
2 weeks and annually for 3 years	Follow-up	Complete resolution at 2 weeks; no recurrence during three annual telephone follow-up contacts.	Annual routine telephone follow-up for former inpatients.

APC = argon plasma coagulation, EMR = endoscopic mucosal resection, IMA = inferior mesenteric artery, VAS = visual analog scale.

## 3. Discussion

This case illustrates third lumbar transverse process syndrome presenting predominantly as acute abdominal pain. The diagnostic process appropriately prioritized life-threatening and organ-specific causes. However, lower-back radiation and movement-related aggravation did not initially lead to targeted palpation and provocation testing at the L3 transverse process. The later diagnosis was supported by focal L3 tenderness and soft-tissue tension, a compatible mechanical pain pattern, and the absence of another disorder sufficient to explain the presentation.

An important lesson from this case is that history taking should be regarded as an iterative diagnostic process rather than a task completed only at the initial encounter. Lower-back pain and radiation to the left lumbar region had been documented from an early stage of the illness, but their diagnostic significance was initially overshadowed by the severity and abdominal location of the presenting pain. When repeated visceral, urinary, and vascular investigations failed to identify a disorder sufficient to explain the symptoms, the clinical history needed to be revisited, with particular attention to the temporal sequence of symptoms, pain radiation, and its relationship to posture and movement. The absence of overt trauma or a single memorable precipitating event should not exclude a musculoskeletal disorder, because sustained or repetitive mechanical loading and prolonged unfavorable posture may not be recognized by the patient as an injury. Reconstructing the history in this manner prompted a focused examination of the left L3 transverse process and helped establish the relationship between the abdominal symptoms and the lumbar findings. Therefore, in patients with persistent unexplained abdominal pain, revisiting the history and performing a targeted physical examination may be more informative than simply adding further investigations.

The initial orthopedic assessment did not attribute the pain to a spinal source on the basis of lumbar MRI. This is not inherently contradictory: mild degenerative MRI findings are common, and routine MRI may neither confirm nor exclude a painful soft-tissue lesion around a transverse process. Lumbar radiography at Wangjing Hospital subsequently demonstrated lumbar scoliosis and degenerative changes. The radiographs remained clinically important because they documented lumbar alignment and osseous degenerative change and complemented, rather than duplicated or replaced, MRI. Conversely, neither modality established the symptomatic pain generator by itself. A subsequent focused examination at Wangjing Hospital elicited localized L3 findings. Lumbar radiography, MRI, the pain pattern, mechanical provocation, and site-specific examination should therefore be interpreted together.^[3,4]^

The relatively long L3 transverse process and its muscular, tendinous, and fascial attachments create a site at which repetitive traction and shear may concentrate. Local tissue injury may generate lumbar pain and referred symptoms through thoracolumbar fascial continuity and convergent sensory input. This referral pattern has a recognized neurophysiological basis. Somatic and visceral nociceptive afferents converge onto common second-order neurons in the dorsal horn of the spinal cord, so that nociceptive input from a deep somatic structure can be perceived in a remote somatic or visceral territory within the same segments. This is the convergence–projection mechanism underlying referred pain.^[8–10]^ Radiography may demonstrate asymmetry or reactive change, and MRI or musculoskeletal ultrasonography may provide supplementary information; however, the diagnosis remains clinical and requires exclusion of urgent alternative causes.^[3,4,11]^

Symptoms improved during a multimodal regimen incorporating Tuina, two acupotomy procedures, and adjunctive medication. Randomized clinical studies have reported short-term improvements in soft-tissue tension, pain, and functional scores after acupotomy-based treatment compared with electroacupuncture or other interventions.^[12,13]^ These studies are limited by setting, sample size, and intervention heterogeneity and do not establish that acupotomy was the principal driver of improvement in this case. Experimental work provides a possible anti-inflammatory mechanism but cannot be directly extrapolated to clinical efficacy.^[14,15]^

The mesenteric arterial findings required careful interpretation. CTA showed mild SMA stenosis and focal proximal IMA stenosis, but distal filling was preserved, mesenteric ultrasonography showed patent flow in the visualized vessels, and vascular consultation concluded that IMA stenosis was unlikely to explain the symptoms. These features reduced, but did not replace, the need for urgent exclusion of mesenteric ischemia and other abdominal emergencies.

Several limitations of this report should be acknowledged. First, pain intensity was documented with the Visual Analog Scale (VAS) only at the emergency presentation, and validated outcome measures such as the VAS and the Oswestry Disability Index were not systematically recorded at discharge or during follow-up; the outcome assessment therefore relies mainly on the patient’s subjective report and physical examination findings. Second, the multimodal regimen combining Tuina, acupotomy, and adjunctive medication precludes attribution of the improvement to any single component. Third, the acupotomy procedures were performed under palpation guidance without ultrasound. As with any blind procedure, this carries the risk that the target tissue may not be accurately addressed, and ultrasound-guided techniques may improve accuracy and safety in future practice. Finally, as a single case report, the favorable three-year outcome cannot be generalized and may partly reflect the natural course of the condition.

## 4. Conclusion

Acute abdominal pain is an uncommon clinical manifestation of third lumbar transverse process syndrome. A thorough evaluation is required to exclude other causes of abdominal pain and to establish a diagnosis through rigorous physical examination. Manual therapy combined with acupotomy treatment may be a potential therapeutic approach for managing third lumbar transverse process syndrome.

## Acknowledgments

We thank the patients for their trust in us.

## Author contributions

**Project administration:** Guangcheng Wei, Ningjuan Yan, Guodong Zhao, Tao Liu, Ming Chen, Weikai Qin, Yong Zhao.

**Writing – original draft:** Guangcheng Wei.

**Writing – review & editing:** Yong Zhao.
